# Molecular Mapping of QTLs Conferring Fusarium Head Blight Resistance in Chinese Wheat Cultivar Jingzhou 66

**DOI:** 10.3390/plants9081021

**Published:** 2020-08-12

**Authors:** Qing Xu, Fuchao Xu, Dandan Qin, Meifang Li, George Fedak, Wenguang Cao, Lijun Yang, Jing Dong

**Affiliations:** 1 Hubei Key Laboratory of Food Crop Germplasm and Genetic Improvement, Institute of Food Crops, Hubei Academy of Agricultural Sciences, Wuhan 430064, China; xuqinghbaas@163.com (Q.X.); xfc2008xfc@163.com (F.X.); hnqdd@163.com (D.Q.); limeifang100@126.com (M.L.); 2 Ottawa Research Development Centre, Agriculture and Agri-Food Canada, Ottawa, ON K1A 0C6, Canada; george.fedak@agr.gc.ca (G.F.); wenguang.cao@agr.gc.ca (W.C.); 3 Hubei Key Laboratory of Crop Disease, Insect Pests and Weeds Control, Institute of Plant Protection and Soil Science, Hubei Academy of Agricultural Sciences, Wuhan 430064, China

**Keywords:** *Fusarium graminearum*, head scab, QTL mapping, *Triticum aestivum*

## Abstract

Fusarium head blight (FHB) is a destructive disease of wheat (*Triticum aestivum* L.), which not only significantly reduces grain yield, but also affects end-use quality. Breeding wheat cultivars with high FHB resistance is the most effective way to control the disease. The Chinese wheat cultivar Jingzhou 66 (JZ66) shows moderately high FHB resistance; however, the genetic basis of its resistance is unknown. A doubled haploid (DH) population consisting 209 lines was developed from a cross of JZ66 and Aikang 58 (AK58), a FHB susceptible wheat cultivar, to identify quantitative trait loci (QTL) that contribute to the FHB resistance. Five field experiments were established across two consecutive crop seasons (2018 and 2019) to evaluate the DH lines and parents for FHB response. The parents and DH population were genotyped with the wheat 55K single-nucleotide polymorphism (SNP) assay. Six QTLs associated with FHB resistance in JZ66 were mapped on chromosome 2DS, 3AS, 3AL, 3DL, 4DS, and 5DL, respectively. Four of the QTL (*QFhb.hbaas-2DS*, *QFhb.hbaas-3AL*, *QFhb.hbaas-4DS*, and *QFhb.hbaas-5DL*) were detected in at least two environments, and the QTL on 3AL and 5DL might be new. The QTL with major effects, *QFhb.hbaas-2DS* and *QFhb.hbaas-4DS*, explained up to 36.2% and 17.6% of the phenotypic variance, and were co-localized with the plant semi-dwarfing loci *Rht8* and *Rht-D1.* The dwarfing *Rht8* allele significantly increased spike compactness (SC) and FHB susceptibility causing a larger effect on FHB response than *Rht-D1* observed in this study. PCR–based SNP markers for *QFhb.hbaas-2DS*, *QFhb.hbaas-3AL*, *QFhb.hbaas-4DS*, and *QFhb.hbaas-5DL*, were developed to facilitate their use in breeding for FHB resistance by marker-assisted selection.

## 1. Introduction

Fusarium head blight (FHB), mainly caused by *Fusarium graminearum,* is one of the most destructive diseases of bread wheat, especially in warm and humid regions [1]. In epidemic years, FHB can cause severe losses in yield and grain quality [2]. Moreover, *Fusarium* mycotoxin contamination renders grain unsuitable for human and animal consumption [3]. The occurrence of FHB has increased in recent years due to climate change, retention of maize and rice stubble, and reduced tillage [4,5]. The disease can be managed by agronomic practices and chemicals to some extent; however, FHB-resistant cultivars are the most cost-effective and environmentally benign way to control the disease and reduce mycotoxin contamination.

Breeding FHB-resistant cultivars depends on the use of resistant germplasm. Numerous elite wheat genotypes with high or moderate resistance have been identified worldwide, including Sumai 3 and Wangshuibai in China [6,7], Chokwang in Korea [8], Ernie in the US [9], Frontana in Brazil [10], and Arina in Switzerland [11]. These resistant sources have greatly facilitated breeding for FHB resistance.

Genetic dissection of FHB resistance has identified hundreds of quantitative trait loci (QTL) distributed across all wheat chromosomes [3,4,12]. A QTL meta-analysis assigned 556 QTL related to FHB resistance into 65 meta-QTLs [13]; however, only a few have been verified to exert major effects on FHB resistance, whereas most have only minor effects [14]. *Fhb1* on 3BS is the most reliable and widely used gene in FHB resistance breeding. Introgression of *Fhb1* into locally adapted wheat cultivars in many breeding programs has demonstrated significant improvements in FHB resistance [15,16,17]. Recently, *Fhb1* was cloned as *His*, encoding a histidine-rich calcium-binding protein [18,19]. The diagnostic marker for *Fhb1* largely facilitates the application of this gene [20]. Pyramiding multiple QTL/genes, including *Fhb1,* is an effective way to enhance FHB resistance in wheat [21]. So far, ten QTL on chromosomes 2B (2), 2D (2), 3A, 3B (*Fhb1*), 3B-2, 4B (*Fhb4*), 5A (*Fhb5*), 6A (2), 6B (*Fhb2*), and 7B have been validated or used in MAS breeding [4].

Jingzhou 66 (JZ66) is a spring wheat cultivar, released in 1975 in Hubei province, which is located in the Middle and Lower Yangtze River Valley in China where FHB epidemics frequently occur. It combines moderately high FHB resistance with good agronomic traits. It was once widely planted in FHB-prevalent regions. The diverse parentage of JZ66 (“Funo/Egypt durum wheat” F_3_//”Mentana/Jingzhou Rye” F_6_) indicates a broad origin. However, the FHB resistance in JZ66 was unlikely to be *Fhb1*, based on pedigree and analysis with an *Fhb1* diagnostic marker in our previous studies [22]. This study aimed to identify the genetic loci responsible for FHB resistance in JZ66 using QTL analysis of a JZ66 derived bi-parental population.

## 2. Results

### 2.1. FHB Phenotypic Variation and Traitcorrelations

**Figure 1:**
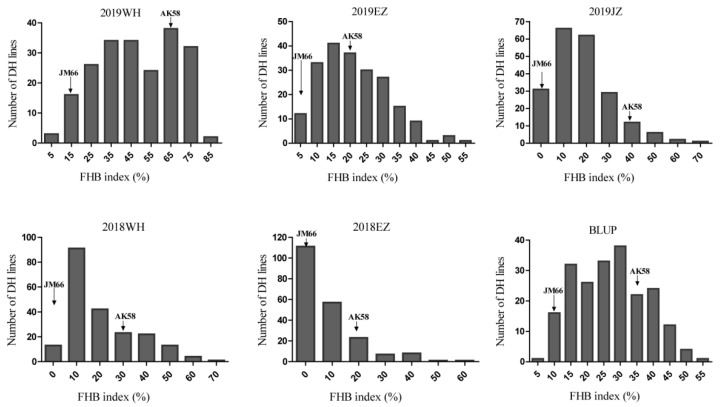
Frequency distribution of FHB index for 209 doubled haploid lines derived from Jingzhou66 and Aikang 58. All data are means of two replicates for each environment. Arrows indicate the disease severities of the parents. WH, Wuhan; EZ, E’zhou; JZ, Jingzhou; best linear unbiased prediction (BLUP).

The FHB severities of the two parents differed significantly, with the resistant parent JZ66 exhibiting a much lower FHB index than Aikang 58 (AK58) across all seasons and locations (Table 1). JZ66 had an FHB index ranging from 3.4% to 19.3%, with an average of 8.5%, whereas AK58 had FHB indices ranging from 21.7% to 68.2%, with an average of 36% (Table 1). FHB index of the doubled haploid (DH) population varied among different environments as well. The FHB index of the population ranged from 0.3% to 81.1% over the five environments. The frequency distribution of FHB index in the DH population was continuous in all environments and the best linear unbiased prediction (BLUP) value across environments, suggesting the quantitative nature of the FHB resistance (Figure 1).

The ANOVA showed significant variation for genotype, environment, and genotype by environment interactions (Table 2). Broad-sense heritability was estimated at 0.78 (Table 2). Correlations among the different environments were highly significant, ranging from 0.21 (*p* < 0.01) between 2018EZ and 2019EZ to 0.65 (*p* < 0.01) between 2019WH and 2019EZ (Table 3).

Correlation analysis revealed negative correlation between FHB severity and plant height, positive correlation between FHB severity and spike compactness, and no significant correlation between FHB severity and days to flowering (Table 4).

### 2.2. Linkage Map Construction

A total of 4500 polymorphic markers, represented by 718 BIN markers, generated 29 linkage groups for the 21 wheat chromosomes. The total map length was 2489.3 cM with an average locus density of 3.5 cM (Supplemental Table S1). Two linkage groups were generated for chromosomes 1A, 1D, 2D, 3D, 5B, 6A, 6B, and 6D. The B genome had the most BIN markers (271, 37.7%), followed by the A genome (36.4%) and D genome (25.7%) (Supplemental Table S2).

### 2.3. QTL for FHB Resistance

**Figure 2:**
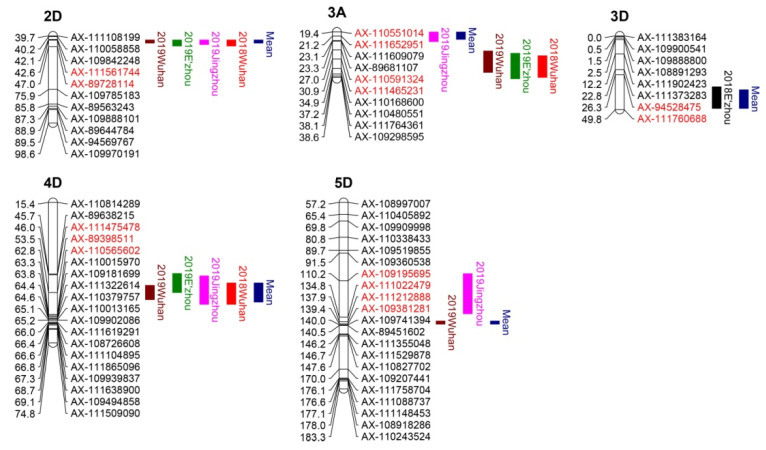
Chromosome locations of quantitative trait loci (QTL) for FHB resistance were detected in one or more environments. The positions of marker loci are shown on the right of the linkage groups and centiMorgan (cM) distances between loci are shown along the left. The environments where the QTL were detected are shown in different colors on the right of the linkage groups. The flanking markers of each QTL are highlighted in red color.

Six QTL for FHB resistance were detected on chromosomes 2DS, 3AS, 3AL, 3DL, 4DS, and 5DL (Figure 2, Table 5). The favorable alleles were all contributed by JZ66. Four QTL on 2DS, 3AL, 4DS, and 5DL were significant in at least two environments and considered stable. QTL on 2DS and 4DS were the major FHB QTL in this population, detected in four environments and datasets of the mean values in all environments. *QFhb.hbaas-2DS* explained 12.3–36.2% of the phenotypic variation with LOD values ranging from 7.8 to 34.4. *QFhb.hbaas-4DS* explained 5.1–17.6% of the phenotypic variation. The physical position of the closely linked markers (*AX-111561744* and *AX-89398511*) to *QFhb.hbaas-2DS* and *QFhb.hbaas-4DS* revealed that they were in the same region as the reduced height genes *Rht8* and *Rht-D1 (Rht2)*, respectively. *QFhb.hbaas-3AL* was detected in three environments, explaining 6.6–8.0% of the phenotypic variation. *QFhb.hbaas-5DL* was significant in 2019WH, 2019JZ, and the dataset of the mean of all the environments, explaining 7.0–9.1% of the phenotypic variation. The remaining two QTL on 3AS and 3DL were each detected in a single environment and the dataset of the mean of all the environments. *QFhb.hbaas-3AS* was detected in 2019JZ and the dataset of the mean value of the FHB index across the environments, explaining 2.9–6.6% of the phenotypic variation. *QFhb.hbaas-3DL* was significant in the 2018EZ environment and dataset of the mean value of all the environments, explaining 2.6–6.4% of the phenotypic variation. Therefore, even though there are phenotypic variation, large effect QTL is more stable than minor effect QTL, and is often detected in more environments.

### 2.4. Effects of QTL on FHB Response

The DH lines were grouped according to the genotypes of the most closely linked markers (*AX-111561744*, *AX-89398511*, *AX-110591324,* and *AX-109381281*) of the four stable QTL (*QFhb.hbaas-2DS, QFhb.hbaas-3AL, QFhb.hbaas-4DS,* and *QFhb.hbaas-5DL*) to evaluate the effects of single QTL and various combinations of the QTL on FHB response. DH lines carrying resistance alleles to all four QTL had much lower FHB indices (reduced by 78%) than lines with no favorable allele. Similarly, lines containing one, two or three favorable QTL had lower FHB indices (reduced by 47.8–69.8%, 54–74%, and 65.1–79.4%) than those with no favorable allele (Table 6). The results suggested that the four stable QTL played an additive role in contributing to FHB resistance. However, difference of the average FHB index between different groups varied due to QTL effect. For example, the average FHB indices of the major effect QTL on 2D and 4D were significantly lower (LSD, α = 0.05) than those of QTLs on 3A and 5D, while there was no significant difference between 3A and 5D (Table 6).

### 2.5. QTL for Plant Height and Spike Compactness

The QTL for plant height and spike compactness were mapped, as they both had a significant correlation with FHB severity in this population. Eight QTLs for plant height, repeatedly detected across the environments, were located on chromosomes 2BL, 2DS, 3DL, 4DS, 5AL, 6BL, 7AL, and 7DL (Table 7). Alleles from AK58 conferred plant height reduction for all the mapped QTL; *QPH.hbaas*-2DS, *QPH.hbaas-4DS*, and *QPH.hbaas-5AL* had major effects on plant height. *QPH.hbaas-2DS* and *QPH.hbaas-4DS* were localized in the same chromosomal region as *Rht8* and *Rht-D1,* with *QPH.hbaas-4DS* having a much larger effect (PVE: 50.36%) on plant height reduction than *QPH.hbaas-2DS* (PVE: 13.73%). These two QTL were coincident with QTL (*QFhb.hbaas-2DS* and *QFhb.hbaas-4DS*) for FHB resistance.

Six stable QTL were detected for spike compactness on chromosomes 2DS, 2DL, 5AL, 5BL, 5DL, and 7AL. All but *QSC.hbaas.2DL* and *QSC.hbaas.5BL* decreased spike compactness and all were derived from JZ66 (Table 7). *QSC.hbaas.2DS* was identical to *QPH.hbaas-2DS* and *QFhb.hbaas-2DS* in position, indicating the pleiotropic effect of this locus (Table 5 and Table 6). *QSC.hbaas.5DL* overlapped with *QFhb.hbaas.5DL* (Table 5 and Table 7).

### 2.6. Development of PCR-Based Single-Nucleotide Polymorphism (SNP) Markers

**Figure 3:**
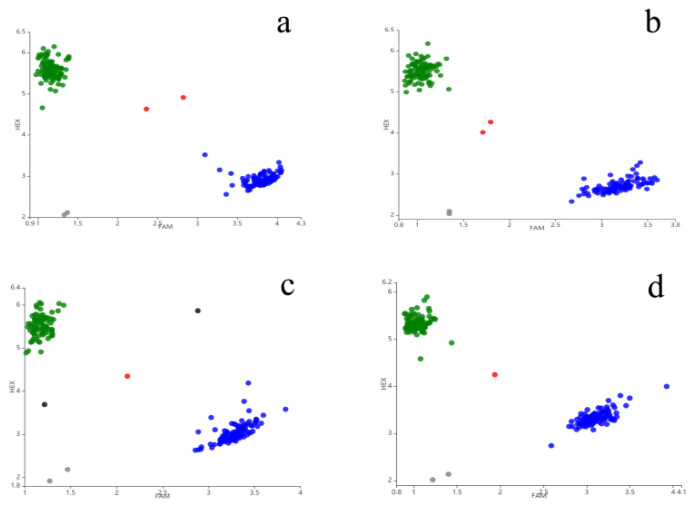
Profiles of four PARMS markers for mapped FHB resistance loci. (**a**) *PARMS-AX-111561744*, *QFhb.hbaas-2DS*, green dots: Susceptible, and blue dots: Resistant; (**b**) *PARMS-AX-110591324*, *QFhb.hbaas-3AL*, green dots: Susceptible, and blue dots: Resistant; (**c**) *PARMS-AX-89398511*, QFhb.hbaas-4DS, green dots: Resistant, and blue dots: Susceptible; and (**d**) *PARMS-AX-109381281*, *QFhb.hbaas-5DL*, green dots: Resistant, and blue dots: Susceptible.

To facilitate QTL application, PARMS markers were developed for SNPs flanking the FHB resistance QTL of *QFhb.hbaas-2DS, QFhb.hbaas-4DS, QFhb.hbaas-3AL,* and *QFhb.hbaas-5DL*. The primer sequences are listed in Table 8, and protocols for their use are described in Supplemental Table S3. For validation, these markers were used to genotype the 209 DHs; the results showed a very low frequency of inconsistency with the original SNP probe (4.3–6.7%). For all four pairs of penta-primer amplification refractory mutation system (PARMS) markers, the SNPs could be differentiated into two main clusters in the DH population (Figure 3).

## 3. Discussion

### 3.1. FHB Assessment and Traitcorrelations

Due to high humidity and warm climate in conditions during the flowering stage, FHB is a frequent occurrence in the Middle and Lower Yangtze River Valley region that includes Hubei province. Numerous wheat cultivars such as Wuhan 1, grown in the region have moderate FHB resistance [23]. JZ66 was identified to have moderate FHB resistance in our previous testing programs. In this study, we undertook five field experiments using a DH population derived from a cross between JZ66 and a highly susceptible wheat cultivar AK58 to investigate the underlying genetic basis for the FHB resistance in JZ66. High correlations among phenotypic data in the different experiments suggested that the results were reliable for QTL analysis. Variations in the frequency distribution pattern indicated that FHB resistance in JZ66 was controlled by quantitative traits. FHB severity was negatively correlated with plant height and positively correlated with spike compactness, indicating that taller plants and plants with less dense spikes had a lower incidence of FHB. Thus, FHB resistance QTL *QFhb.hbaas-2DS* and *QFhb.hbaas-4DS*, were co-localized with plant height QTL *QPH.hbaas-2DS* and *QPH.hbaas-4DS*. FHB resistance QTL, *QFhb.hbaas-2DS* and *QFhb.hbaas-5DL*, also overlapped with QTL for spike compactness (*QSC.hbaas-2DS* and *QSC.hbaas-5D*L).

### 3.2. Novelty of Mapped QTL for FHB Resistance

The first stable QTL identified for FHB resistance was located on chromosome 2DS. Alleles from JZ66 were associated with enhanced FHB resistance. Several previous studies reported FHB resistance related QTL in the same chromosome region. In a DH mapping population derived from Sumai 3 and Gamenya, Xu et al. (2001) [24] reported a QTL on 2DS, with resistance closely associated with the *Xgwm261* allele in the susceptible parent Gamenya. The same QTL was also detected in a RIL population developed from Ning 894,037 and Alondra, with resistance contributed by the moderately susceptible parent Alondra [25]. It is noteworthy that Ning 894,037 derived from Italian cultivar Funo, was one of the parents of Sumai 3. Using comparative analysis, Handa et al. (2008) [26] identified a 2DS region responsible for FHB resistance and validated the locus in the Sumai 3 and Gamenya population. This QTL was also detected in the Haicandou and Jagger population, with the resistance allele on 2DS from susceptible Jagger [27]. In the studies mentioned above, all of the QTL were linked to the marker *Xgwm261*, indicating that they are likely the same QTL. In all cases, the FHB resistance allele on 2DS was conferred by the susceptible parent, particularly in Sumai 3-derived populations. Interestingly, Basnet et al. (2012) [28] identified an FHB susceptible locus on 2DS from Sumai 3, linked with marker *Xgwm261* in a Sumai 3 and Y119306 (highly susceptible Tibetan landrace) population. It is well-known that *Xgwm261* is the diagnostic marker for the semi-dwarf gene *Rht8*; therefore, the QTL identified for FHB resistance on chromosome 2DS might be due to the effect of *Rht8.* Our laboratory data and previous studies [29,30] showed that Sumai 3 has the 192 bp size band for *Xgwm261*, indicating that it likely contains the dwarfing allele of *Rht8* and probably inherited this gene from its Funo parent (Bai et al. 2004). However, in these abovementioned published studies, plant height data were not available for further comparisons. In our study, the closely linked marker *(AX-111561744)* to *QFhb.hbaas-2DS* is located at 23.4 Mb, close to *Xgwm261* (19.6 Mb). Analysis of *Xgwm261* indicated that JZ66 did not carry *Rht8*, whereas AK58 did (unpublished data). Therefore, we speculate that the taller *Rht8* allele in JZ66 contributes its FHB resistance. Similar results were obtained in an YZ1 and NX188 RIL population. *QFHB.caas-2D* and *QPH.caas-2D* were simultaneously identified at the position of *Rht8*, the FHB-resistance allele contributed by YZ1 and the plant height reducing allele was from NX188 [31].

The second stable QTL, with a large effect on FHB response mapped in this study, was located on chromosome 4DS (*QFhb.hbaas-4DS*). The favorable allele of this QTL for FHB resistance was derived from JZ66 and co-localized with the plant height gene *Rht-D1* (also known as *Rht2*) by physical position comparison between the closely linked marker *AX-89398511* of *QFhb.hbaas-4DS* (17.6 Mb) and *Rht-D1* (19 Mb). This QTL was also detected for plant height, and the allele for reduced plant height was from AK58. It has long been known that the *Rht-D1* locus is associated with FHB resistance. Draeger et al. (2007) [32] detected a stable FHB resistance QTL on 4DS co-localized with *Rht–D1,* and the *Rht-D1a* allele from the Swiss cultivar Arina contributed resistance. In a Spark and Rialto DH population, the reduced height allele *Rht-D1b* increased susceptibility to FHB [33]. Investigation of the effect of *Rht-D1* on FHB rating in three segregating populations revealed that *Rht-D1b* reduced plant height by 7–18%, but increased FHB incidence by 22–53% [34]. The authors concluded that the coincidence of QTL for FHB and plant height was not due to plant height per se but to either linked genes conferring FHB susceptibility in the same interval and/or a pleiotropic physiological effect of the dwarfing allele enhancing susceptibility.

*QFhb.hbaas-3AL* was detected in three environments. The physical position of the closely linked marker *AX-110591324* to *QFhb.hbaas-3AL* was around 530 Mb. In previous reports, a major effect QTL associated with FHB resistance on 3AL was identified in Brazilian cultivar Frontana, near marker *Xdupw227* (598 Mb) [10]. This QTL was confirmed in a Frontana and Seri82 population [35]. In an Arina and Forno population, Paillard, et al. (2004) [11] reported a minor effect 3AL QTL flanked by markers *Xwmc264* and *Xgwm155* (625–702 Mb). However, the physical positions of these markers were not close to *QFhb.hbaas-3AL.* We therefore speculated that the *QFhb.hbaas-3AL* identified in JZ66 was different from those reported in previous studies.

The fourth stable QTL was designated as *QFhb.hbaas-5DL*, and was detected in two environments and combined mean, explaining 2.7–11.5% of the phenotypic variances. Lv et al. (2014) [31] reported an FHB resistance QTL on 5DL (*QFHB.caas-5D*), flanked by markers *Xgwm292* and *Vrn-D1*, with a PVE of 12.8%. A 5DL QTL near marker *Xgwm292* in the Wangshuibai and Wheaton population contributed three types (type I–III) of FHB resistance [36]. A minor QTL for FHB resistance on 5DL in the CIMMYT spring wheat Catbird, peaked at *Vrn-D1* [37]. The abovementioned QTL might be the same locus as both were close to *Xgwm292,* which is adjacent to *Vrn-D1*. The physical position of the closely linked marker (*AX-111212888*) to *QFhb.hbaas-5DL* was 494 Mb, which is 26 Mb from marker *Xgwm292* (468 Mb). Therefore, *QFhb.hbaas-5DL* is likely to be new.

### 3.3. Coincidence of QTL for FHB Response and Other Traits

Co-localization of QTL for FHB resistance and plant height has been found in numerous mapping populations [10,32,33,38], but not all plant height QTL were simultaneously associated with FHB response. In the current study, two major QTL (*QFhb.hbaas-2DS* and *QFhb.hbaas-4DS*) for FHB resistance coincided with reduced height QTL *QPH.hbaas*-2DS and *QPH.hbaas-4DS*. Both favorable alleles for FHB resistance were associated with tallness. The physical positions of these loci were most likely the same as the height genes *Rht8* on 2DS and *Rht-D1 (Rht2)* on 4DS. *QPH.hbaas-4DS* had a much larger effect on reducing plant height than *QPH.hbaas-2DS*, as reported previously [39]. The effect of plant height genes on FHB resistance does not appear to be due to plant height per se, as some other identified QTL for plant height did not coincide with QTL for FHB response. However, we noticed that these two loci had an adverse effect on FHB response, with the effect of *QFhb.hbaas-2DS* (mean PVE: 36.2%) on FHB resistance much larger than that of *QFhb.hbaas-4DS* (mean PVE: 14.5%). The favorable alleles from JZ66 decreased FHB severity by 69.9% for *QFhb.hbaas-2DS*, and 55.5% for *QFhb.hbaas-4DS*.

The QTL with the largest effect on spike compactness (*QSC.hbaas.2DS)* was identical to the QTL for FHB resistance and plant height at the position of *Rht8*. Alleles from JZ66 reduced spike compactness by 16%, relative to alleles from AK58 (Supplemental Table S4). It was concluded that the allelic effects from JZ66 at the 2DS locus were to increase FHB resistance and plant height, but to decrease the spike compactness (Supplemental Table S4). A similar QTL coincidence was found for *QFhb.hbaas-5DL* and *QSC.hbaas.5DL*. Both QTL affected FHB resistance and decreased spike compactness, and the favorable allele for resistance was from JZ66 (Supplemental Table S4). These results suggest that QTL contributing to decreased spike compactness, could reduce the rate of spread of fungal colonization, and therefore a mechanism for enhanced FHB resistance in JZ66. Interestingly, cultivars from the Middle and Lower Yangtze River Valley generally have low spike compactness compared with cultivars from the more northern Yellow and Huai River Valley wheat region. This hints that the low spike compactness might be a selection target in breeding for FHB resistance, especially in FHB epidemic regions. The effect of the dwarfing allele of *Rht8* on increasing spike compactness might explain its greater reduction of FHB resistance than *Rht-D1b*. Thus, the utilization of *Rht8* should be avoided in breeding for FHB resistance. In agreement with this, a lower frequency of cultivars with *Rht8* were reported in cultivars (more FHB resistant) from the Middle and Lower Yangtze River Valley compared to cultivars from Northern wheat regions (more FHB susceptible) [40,41].

### 3.4. Perspectives on Breeding for FHB Resistance

Sumai 3 has been identified as the most FHB-resistant cultivar and is exploited worldwide in wheat FHB breeding. However, the poor agronomic performance of Sumai 3 and its progeny hampers its use in China. Moderately resistant cultivars often have multiple QTL with small or medium effects and tend to be more adapted. Therefore, a breeding strategy using cultivars with better yield and moderate FHB resistance was proposed by Cheng et al. (2003) [42] and was used to develop numerous cultivars with high yield and moderate FHB resistance, including Yangmai 158, Yangmai 11, and Yangmai 16 [43,44]. These cultivars were widely planted in Middle and Lower Yangtze River Valley, and have made significant contributions to Chinese wheat production. A similar strategy was used in Hubei province to develop wheat cultivars, including JZ66, Emai 18 and Emai 006, with higher yield and much improved FHB resistance. However, the FHB resistance source of these cultivars differed from that of the Yangmai series (mainly developed for Jiangsu province). Therefore, in terms of long-term breeding, introgression of *Fhb1* into local wheat cultivars through backcrossing will significantly improve FHB resistance in Hubei province. Recently, Zhu et al. [23] proposed that Ningmai 9 and its derivates contained *Fhb1* but with good agronomic traits, providing better parental material for incorporating *Fhb1* in local breeding populations. Alternatively, FHB resistance of wheat cultivars can be improved by stacking resistant loci from local cultivars, such as those discovered in the present study. In particular, the PCR-based markers developed in this study will facilitate the use of FHB resistance in JZ66.

## 4. Materials and Methods

### 4.1. Plant Materials

A doubled haploid population of 209 lines was developed from a cross between JZ66 and FHB-susceptible cultivar AK58. AK58 was a leading cultivar in the Yellow and Huai River Valley in China with semi-dwarf plant height. Wheat cultivars Sumai 3, E’en 1, and An’nong 8455 were used as resistant, moderately resistant, and susceptible checks in all the experiments.

### 4.2. Evaluation of FHB Reaction and Measurement of Agronomic Traits

The DH lines and parents, along with the checks, were evaluated for FHB reaction in five environments across two cropping seasons (2017–2018 and 2018–2019) and three locations, Wuhan, E’zhou, and Jingzhou. The trials were abbreviated as 2018WH, 2018EZ, 2019WH, 2019EZ, and 2019JZ. All trials were arranged in randomized complete blocks with two replicates. Each plot consisted of single 1 m rows spaced 25 cm apart, and sown with approximately 60 seeds.

Experiments at Wuhan were in an FHB screening nursery equipped with an overhead misting system to facilitate disease development. Plots were misted hourly for 2 min from 9 am to 7 pm daily from inoculation to completion of disease assessment. At anthesis, ten flowering spikes were randomly selected in each plot, labeled with blue 3 M tape, and sprayed with ~30 mL a water suspension of conidiospores of two isolates *F. graminearum* collected from Huanggang and Wuhan, Hubei province. The suspension contained 1 × 10^5^ spores mL^−1^ and was amended with 0.01% Tween 20 detergent. The FHB responses of the ten labeled spikes were scored by counting the number of infected and total spikelets at 21 d after inoculation. The FHB index (%) was calculated as FHB incidence times severity, where incidence was the average percentage of infected spikes, and severity was the average percentage of symptomatic spikelet [45].

Inoculation was performed at E’Zhou and Jingzhou, by scattering 600 g scabby wheat grains per 100 m^2^ on the soil surface about three weeks before anthesis. The FHB incidence and severity were evaluated at 28 d post anthesis by counting infected spikes and infected spikelets per spike on 30 spikes in each plot.

Plant height, days to flowering, spike length, and total spikelets per spike were also measured. Spike compactness was calculated by dividing the number of spikelets per spike by spike length. Days to flowering were recorded as the number of days from sowing to anthesis. Plant height was measured from the ground to top of the spike (awns excluded) at maturity. Spike length was measured from the base to the top of the spike, not including the awns.

### 4.3. Statistical Analysis

The calculations for descriptive statistics, correlation analysis, and analysis of variance (ANOVA) were performed using SPSS (IBM SPSS Statistics, Chicago, IL, USA). The frequency distribution for the FHB index at each site and BLUP value for all sites was produced using Origin programs (OriginLab, Northampton, MA, USA). The BLUP value was calculated with the R package Lme4 (y = lmer (Trait ~ (1|Genetype) + (1|Year)). Broad-sense heritability (*H*^2^) was calculated from the ANOVA using the following formula: *H*^2^ = 1 − *MS*_2_*/MS*_1_, where *MS*_1_ and *MS*_2_ are the mean squares for genotype and genotype by environment interaction, respectively [46]. Mean values of two replicates at each environment were used for statistical analysis and QTL mapping.

### 4.4. Genotyping

Genomic DNA was extracted from fresh leaves of DH lines and parents with a Plant Genomic DNA Kit (Tiangen Bio-tech, Beijing). DNA integrity was checked on agarose gel, and the quantity was measured with a Nanodrop spectrometer. The population was genotyped using the wheat 55 K SNP array [47] that contains 53,063 markers, according to the Affymetrix Axiom 2.0 Assay Manual Workflow protocol by China Golden Marker Biotech Co. Ltd. (Beijing). SNP probes that were categorized as PolyHigh-Resolution (15,978 markers) were kept. The SNP filtering criteria were set as follows: Monomorphic SNPs, those showing call rates less than 80%, and sites with significantly distorted segregation ratios (*p* < 0.05) were removed from the dataset.

### 4.5. Genetic Map Construction

The filtered SNPs (4500) were analyzed to eliminate redundant markers using the BIN function in IciMapping 4.1 with the parameter “not considering the missing value” [48]. One marker from each bin was randomly selected for genetic map construction. Linkage groups were generated with 718 bin markers using Joinmap V4.0 [49]. Recombination fractions were converted to centiMorgans (cM) using the Kosambi function [50]. The linkage groups were graphically visualized with Mapchart V2.3 [51].

### 4.6. QTL Analysis

Estimates of QTL positions and their effect in each environment were determined by inclusive composite interval mapping of additive function (ICIM-ADD) in IciMapping V4.1 [26,52]. A LOD threshold of 2.5 was set to declare a significant QTL. The effects of the detected QTL were estimated as the phenotypic variance explained by ICIM. QTL situated within the same interval or overlapping confidence regions were considered identical, while those identified in more than one environment were considered stable. To compare the physical positions of the QTL identified in the current study and known QTL, closely linked markers were blasted against the Chinese Spring reference genome sequences (IWGSC RefSeq v1.0) or searched on the website http://202.194.139.32/ to obtain the physical position.

### 4.7. PCR-Based SNP Marker Development

SNPs significantly associated with FHB response were converted to PARMS markers [53] in Wuhan Gentides Biotechnology Co., LTD. (Wuhan, China) (http://www.gentides.com) for use in MAS breeding. Primers for PARMS markers were designed by PolyMarker (http://polymarker.tgac.ac.uk) according to sequences of flanking SNP markers. PCR were performed in 384-well PCR plates for PARMS genotyping. The 5 μL PCR reaction system contained 2 times PARMS PCR reaction mix, 150 nM of each allele-specific primer, 400 nM locus-specific primer, and 1.4 μL alkaline lysis DNA template; 5 μL mineral oil was added into each well to prevent evaporation of the PCR mix. The thermal cycler program of PARMS was denaturing at 95 °C for 15 min, followed by 10 cycles of denaturation at 95 °C for 20 s, annealing started at 65 °C for 1 min, decreasing 0.8 °C per cycle to 57 °C; followed by 32 cycles of denaturation at 95 °C for 20 s and annealing at 57 °C for 1 min. The well plate was read using a TECAN Infinite M1000 plate reader. SNP calling and plots were carried out by an online software snpdecoder (http://www.snpway.com/snpdecoder/) combining manual modification.

## 5. Conclusions

The results from the current study showed that the moderate FHB resistance in JZ66 is controlled by multiple QTL with additive effects. Two QTL on chromosomes 2DS and 4DS played dominant roles in FHB resistance, and co-localized with the reduced height *Rht8* and *Rht-D1 (Rht2)* loci. Co-localization of FHB resistance QTL with the *Rht8* and *Rht-D1* loci but not some other reduced plant height QTL suggested a pleiotropic effect of these two loci. FHB resistance in JZ66 was associated with decreased spike compactness, controlled by *QSC.hbaas.2DS* and *QSC.hbaas.5DL*. The moderate FHB resistance and better agronomic traits thus endowed JZ66 a good germplasm for FHB resistance breeding. The PARMS marker designed for the stable QTL identified in this study may facilitate the pyramiding of resistance gene/QTL.

## Figures and Tables

**Table 1:** Range and mean of Fusarium head blight (FHB) indices of parents and the Jingzhou 66 (JZ66) and Aikang 58 (AK58) doubled haploid (DH) population.

Environment ^a^	Parents		DH Population						
	JZ66	AK58	Max	Min	Mean	SD ^b^	CV ^c^	Skewness	Kurtosis
2019WH	19.3	68.2	81.1	9.5	47.8	19.3	40.3	−0.12	−1.1
2019EZ	4.9	23.6	54.3	5.4	21.1	10.3	48.7	0.7	0.1
2019JZ	4.4	36.6	66.8	1.9	18.1	12.3	67.9	1.1	1.3
2018WH	10.4	30.1	69.0	3.3	20.7	15.1	72.7	1.1	0.2
2018EZ	3.4	21.7	55.2	0.3	8.8	10.2	116.8	1.9	3.7

^a^ WH, EZ, and JZ indicate Wuhan, E’zhou, and Jingzhou, respectively. ^b^ SD, standard deviation. ^c^ CV, coefficient of variation in %.

**Table 2:** Analysis of variance and heritability for FHB index in DH population across two years.

Source of Variation	Df ^a^	Mean Square	F Test	*p* Value	*h* ^2^ ^b^
Environments	4	92,764.23	799.83	<0.001	0.78
Genotypes	208	1328.22	11.45	<0.001	
Genotype × Year	832	297.92	2.57	<0.001	
Error	1043	115.98			

^a^ Df, Degree of freedom. **^b^** h^2^, Heritability.

**Table 3:** Pearson correlation coefficients of the FHB index across different environments.

Environments ^a^	2019WH	2019EZ	2019JZ	2018WH	2018EZ
2019WH	–				
2019EZ	0.65 **				
2019JZ	0.62 **	0.64 **			
2018WH	0.45 **	0.46 **	0.29 **		
2018EZ	0.30 **	0.21 **	0.30 **	0.34 **	

^a^ WH, EZ, and JZ indicate Wuhan, E’zhou, and Jingzhou, respectively. ** indicate significance at *p* < 0.01.

**Table 4:** Correlations between FHB index and plant height (PH), days to flowering (DF), and spike compactness (SC) in the Jingzhou66 × Aikang58 DH population.

	PH	DF	SC
FHB index (%)	−0.62 **	0.11	0.49 **

** indicate significance at *p* < 0.01.

**Table 5:** QTL for FHB resistance identified in the Jingzhou66 and Aikang58 DH population using inclusive composite interval mapping.

QTL	Environment ^a^	Position (Mb)	Marker Interval		LOD ^b^	PVE ^c^	ADD ^d^
*QFhb.hbaas* *-2DS*	2019WH	23.4–26.3	AX-111561744	AX-89728114	28.9	28.0	13.2
	2019EZ	23.4–26.3	AX-111561744	AX-89728114	22.9	30.5	8.0
	2019JZ	23.4–26.3	AX-111561744	AX-89728114	18.3	24.2	7.1
	2018WH	23.4–26.3	AX-111561744	AX-89728114	7.8	12.3	6.2
	Mean	23.4–26.3	AX-111561744	AX-89728114	34.4	36.2	7.3
*QFhb.hbaas* *-3AS*	2019JZ	54.8–67.6	AX-110551014	AX-111652951	2.6	2.9	2.4
	Mean	54.8–67.6	AX-110551014	AX-111652951	8.5	6.6	3.1
*QFhb.hbaas* *-3AL*	2019WH	530.9–617.6	AX-110591324	AX-111465231	6.3	6.6	6.4
	2019EZ	530.9–617.6	AX-110591324	AX-111465231	5.2	8.0	4.1
	2018WH	530.9–617.6	AX-110591324	AX-111465231	3.5	7.4	4.8
*QFhb.hbaas* *-3DL*	2018EZ	536.0–566.1	AX-94528475	AX-111760688	3.4	6.4	2.6
	Mean	536.0–566.1	AX-94528475	AX-111760688	3.5	2.6	2.0
*QFhb.hbaas* *-4DS*	2019WH	17.0–31.3	AX-89398511	AX-110565602	20.0	17.6	10.5
	2019EZ	15.2–17.0	AX-111475478	AX-89398511	4.6	5.1	3.3
	2019JZ	15.2–17.0	AX-111475478	AX-89398511	5.8	6.8	3.8
	2018WH	17.0–31.3	AX-89398511	AX-110565602	7.0	10.9	5.8
	Mean	15.2–17.0	AX-111475478	AX-89398511	16.7	14.5	4.6
*QFhb.hbaas* *-5DL*	2019WH	494.5–495.2	AX-111212888	AX-109381281	11.5	9.1	7.5
	2019JZ	447.9–489.3	AX-109195695	AX-111022479	5.1	7.0	3.8
	Mean	494.5–495.2	AX-111212888	AX-109381281	3.7	2.7	2.0

^a^ WH, EZ, and JZ indicate Wuhan, E’zhou, and Jingzhou, respectively. ^b^ LOD, peak LOD score. ^c^ PVE, phenotypic variation explained (R^2^, %) by each QTL. ^d^ ADD, additive effect. Positive value indicates the allele from AK58 increases the trait value.

**Table 6:** Effect of individual or combined QTL on FHB response.

Number of QTLs	QTL Combination	Mean FHB Index across Environments ^b^	Reduction % ^a^
None	-	49.6a	-
1	2D	15.0d	69.8
	4D	22.1c	55.4
	3A	25.9b	47.8
	5D	24b	51.6
2	2D/4D	12.9e	74.0
	2D/3A	16.8d	66.1
	2D/5D	16.3d	67.1
	4D/3A	18.7cd	62.3
	4D/5D	18.7cd	62.3
	3A/5D	22.8bc	54.0
3	2D/4D/3A	10.2e	79.4
	2D/4D/5D	12.7e	74.4
	2D/3A/5D	13.8de	72.2
	4D/3A/5D	17.3cd	65.1
4	2D/4D/3A/5D	11.1e	77.6

^a^ % reduction was calculated from comparison with lines without favorable alleles of the QTL. ^b^ Fisher’s least significant difference test was used to determine significant differences among means of different groups. The same letters following the mean FHB index value indicate no significant difference at α = 0.05.

**Table 7:** QTL identified for plant height and spike compactness in the Jingzhou66 and Aikang58 DH population using inclusive composite interval mapping.

QTL	Physical Position (Mb)	Marker Interval		LOD ^a^	PVE ^b^	Additive
Plant height						
*QPH.hbaas-2BL*	765.3–769.1	AX-111609703	AX-108792274	4.94	2.48	−2.27
*QPH.hbaas-2DS*	23.4–26.3	AX-111561744	AX-89728114	22.90	13.73	−5.33
*QPH.hbaas-3DL*	518.3–536.0	AX-111373283	AX-94528475	2.74	1.44	−1.59
*QPH.hbaas-4DS*	17.1–31.4	AX-89398511	AX-110565602	66.70	50.36	−10.75
*QPH.hbaas-5AL*	525.2–535.1	AX-111083486	AX-109378942	28.21	12.63	−5.39
*QPH.hbaas-6BL*	439.2–565.7	AX-111578782	AX-108940703	8.60	3.52	−2.50
*QPH.hbaas-7AL*	701.2–701.3	AX-110516258	AX-111683497	4.66	2.25	−2.17
*QPH.hbaas-7DL*	665.4–718.1	AX-111061288	AX-108780423	5.02	1.10	−1.71
Spike compactness						
*QSC.hbaas.2DS*	23.4–26.3	AX-111561744	AX-89728114	45.51	43.61	0.24
*QSC.hbaas.2DL*	523.6–526.2	AX-109419238	AX-111566799	5.05	2.96	−0.06
*QSC.hbaas.5AL*	525.2–535.1	AX-111083486	AX-109378942	11.05	10.10	0.12
*QSC.hbaas.5BL*	546.1–548.7	AX-110593685	AX-111508809	5.37	3.22	−0.06
*QSC.hbaas.5DL*	494.6–495.2	AX-111212888	AX-109381281	2.55	2.01	0.06
*QSC.hbaas.7AL*	671.4–673.8	AX-111536514	AX-110518554	4.78	2.90	0.06

^a^ LOD, peak LOD score. ^b^ PVE, phenotypic variation explained (R^2^, %) by each QTL.

**Table 8:** Primers for penta-primer amplification refractory mutation system (PARMS) markers developed from single-nucleotide polymorphisms (SNPs) associated with four stable FHB resistance loci.

QTL	Marker	Primer	Sequences
*QFhb.hbaas-2DS*	*PARMS-AX-111561744*	A	GAAGGTGACCAAGTTCATGCTCTTTGAGGCAGTCCAGTCCC
		B	GAAGGTCGGAGTCAACGGATTCTTTGAGGCAGTCCAGTCCA
		common	CCTGAGCAACCTAATTCAATAGC
*QFhb.hbaas-4DS*	*PARMS-AX-89398511*	A	GAAGGTGACCAAGTTCATGCTCATTGATCATAGAAACTGCCTCAT
		B	GAAGGTCGGAGTCAACGGATTCATTGATCATAGAAACTGCCTCAC
		common	ACGGATTCATGTGGAGCTTG
*QFhb.hbaas-3AL*	*PARMS-AX-110591324*	A	GAAGGTGACCAAGTTCATGCTGGTCCAACACCTCTCTAAGCGT
		B	GAAGGTCGGAGTCAACGGATTGTCCAACACCTCTCTAAGCGC
		common	ATGAAGGCGAACCAGACGG
*QFhb.hbaas-5DL*	*PARMS-AX-109381281*	A	GAAGGTGACCAAGTTCATGCTGGCGAAGGTATGGTCCAGTC
		B	GAAGGTCGGAGTCAACGGATTGGCGAAGGTATGGTCCAGTT
		common	CTGACGTGGTGACGCCTTT

Tails for competitive primers are underlined.

## Supplementary Materials

The following are available online at https://www.mdpi.com/2223-7747/9/8/1021/s1, Table S1: Protocols for four penta-primer amplification refractory mutation system (PARMS) markers developed from SNPs associated with four mapped FHB-resistant loci; Table S2: Effect of FHB-resistant QTL on plant height and spike compactness.
