# Supplementary material for: Molecular Mapping of QTLs Conferring Fusarium Head Blight Resistance in Chinese Wheat Cultivar Jingzhou 66
Source: Plants (Basel). 2020 Aug 12;9(8):1021. doi: 10.3390/plants9081021 (PMC7465298; doi:10.3390/plants9081021)
Supplement: Supplementary file 1 [file plants-09-01021-s001.pdf]

Supplemental tables

Table 1. Details of markers in the constructed map.

Linkage Groups	Chromosome	Number of Markers	Length（cM）
1	1A_1	10	52.57
2	1A_2	5	7.87
3	1B	37	100.00
4	1D_1	9	21.28
5	1D_2	5	61.07
6	2A	30	60.43
7	2B	27	87.64
8	2D_1	35	150.40
9	2D_2	6	60.02
10	3A	42	121.61
11	3B	39	136.94
12	3D_1	8	49.75
13	3D_2	8	15.26
14	4A	35	147.24
15	4B	23	86.27
16	4D	31	102.63
17	5A	58	173.48
18	5B_2	50	138.43
19	5B_1	5	6.75
20	5D	32	183.31
21	6A_1	17	79.75
22	6A_2	8	16.85
23	6B_1	37	85.67
24	6B_2	5	7.70
25	6D_1	7	4.17
26	6D_2	14	103.05
27	7A	57	189.71
28	7B	48	153.49
29	7D	30	85.99
Total	29	718	2489.29

Table 2. Details of markers on each genome.

A Genome	NO. of Markers	Length (cM)	B Genome	NO. of Markers	Length (cM)	D Genome	NO. of Markers	Length (cM)
1A_1	10	52.57	1B	37	100.00	1D_1	9	21.28
1A_2	5	7.87	2B	27	87.64	1D_2	5	61.07
2A	30	60.43	3B	39	136.94	2D_1	35	150.40
3A	42	121.61	4B	23	86.27	2D_2	6	60.02
4A	35	147.24	5B_2	50	138.43	3D_1	8	49.75
5A	58	173.48	5B_1	5	6.75	3D_2	8	15.26
6A_1	17	79.75	6B_1	37	85.67	4D	31	102.63
6A_2	8	16.85	6B_2	5	7.70	5D	32	183.31
7A	57	189.71	7B	48	153.49	6D_1	7	4.17

						6D_2	14	103.05
						7D	30	85.99
Total	262	849.49		271	802.88		185	836.91
Markers density		3.24			2.96			4.52

Table 3. Protocols for four penta-primer amplification refractory mutation system (PARMS) markers developed from SNPs associated with four mapped FHB-resistant loci.

QTL	PARMS marker	PCR system	PCR program	Detection	Result calling	
					Resistance	Susceptibility
QYr.hbaas-2DS	*PARMS-AX-111561744*	5 μL reaction contains 2x PARMS Master Mix, 150 nM allele-specific primer, 400 nM Locus-specific primer, 1.4 μL alkaline lysis DNA	15 min at 94°C; 10 touchdown cycles with a 0.8°C drop for annealing and elongation per cycle (94°C for 20 s, 65°C for 1 min); 32 cycles (94°C 20 s, 57°C 1 min)	Fluorescence signals were detected using the multifunctional microplate reader TECAN Infinite M1000 and analyzed using online snpdecoder software.	Blue dot	Green dot
QYr.hbaas-3AL	*PARMS-AX-110591324*				Blue dot	Green dot
QYr.hbaas-4DS	*PARMS-AX-89398511*				Green dot	Blue dot
QYr.hbaas-5DL	*PARMS-AX-109381281*				Green dot	Blue dot

Table 4. Effect of FHB-resistant QTL on plant height and spike compactness.

QTL	Allele[a]	Plant height (cm)	Pvalue	Spike compactness	Pvalue
QFhb.hbaas-2DS	R	89.58	P<0.01	2.18	P<0.01
	S	77.55		2.60	
QFhb.hbaas-3AL	R	93.06	P=0.21	2.42	P=0.14
	S	74.07		2.35	
QFhb.hbaas-4DS	R	82.60	P<0.01	2.41	P=0.18
	S	84.99		2.35	
QFhb.hbaas-5DL	R	84.73	P=0.35	2.33	P<0.05
	S	82.95		2.43	

[a] R and S indicate the resistant and susceptible alleles of corresponding QTL, respectively
